# The role of natriuretic peptides in the management, outcomes and prognosis of sepsis and septic shock

**DOI:** 10.5935/0103-507X.20190060

**Published:** 2019

**Authors:** Govind Pandompatam, Kianoush Kashani, Saraschandra Vallabhajosyula

**Affiliations:** 1 Division of Pulmonary and Critical Care Medicine, Department of Medicine, Mayo Clinic - Rochester, Minnesota, United States.; 2 Division of Nephrology and Hypertension, Department of Medicine, Mayo Clinic - Rochester, Minnesota, United States.; 3 Department of Cardiovascular Medicine, Mayo Clinic - Rochester, Minnesota, United States.

**Keywords:** Sepsis, Shock, septic, Natriuretic peptides, Natriuretic peptide, brain, Ventricular dysfunction

## Abstract

Sepsis continues to be a leading public health burden in the United States and worldwide. With the increasing use of advanced laboratory technology, there is a renewed interest in the use of biomarkers in sepsis to aid in more precise and targeted decision-making. Natriuretic peptides have been increasingly recognized to play a role outside of heart failure. They are commonly elevated among critically ill patients in the setting of cardiopulmonary dysfunction and may play a role in identifying patients with sepsis and septic shock. There are limited data on the role of these biomarkers in the diagnosis, management, outcomes and prognosis of septic patients. This review seeks to describe the role of natriuretic peptides in fluid resuscitation, diagnosis of ventricular dysfunction and outcomes and the prognosis of patients with sepsis. B-type natriuretic peptide (BNP) and N-terminal pro-BNP (NT-proBNP) have been noted to be associated with left ventricular systolic and diastolic and right ventricular dysfunction in patients with septic cardiomyopathy. BNP/NT-proBNP may predict fluid responsiveness, and trends of these peptides may play a role in fluid resuscitation. Despite suggestions of a correlation with mortality, the role of BNP in mortality outcomes and prognosis during sepsis needs further evaluation.

## INTRODUCTION

Sepsis remains a major cause of morbidity and mortality, both within the intensive care unit (ICU) and outside, accounting for nearly US$17 billion of annual healthcare expenditures.^(1-5)^ Despite significant improvements in the diagnosis and management of sepsis, it remains a challenging clinical entity due to its varied etiology and presentation.^(3,6-14)^ With the development of sensitive laboratory technology, there is a renewed interest in the use of biomarkers for the targeted treatment of sepsis. To date, 178 biomarkers have been studied in septic patients in recent years, with C-reactive protein and procalcitonin being the most commonly used tests in current clinical practice.^(7)^ Although these biomarkers can aid in diagnosis, determination of the severity and prognosis of sepsis, their specificity, and their prognostic implications have been described with wide variability.^(15,16)^ Cardiac biomarkers have been evaluated previously in these patients to aid in prognosis and in the diagnosis of myocardial injury and/or dysfunction.^(17-19)^ Cardiac troponin-T and I have shown a strong correlation between sepsis severity and outcomes and prognosis.^(18,20)^ However, the role of commonly used natriuretic peptides, namely, B-type natriuretic peptide (BNP) and N-terminal pro-BNP (NT-proBNP), have not been thoroughly studied.^(21)^

This study sought to evaluate the existing literature on the role of BNP/NT-proBNP in the diagnosis and management of sepsis with or without concomitant septic cardiomyopathy. Prior reviews on this topic have focused on outcomes and prognosis in sepsis;^(22)^ however, there are limited summations of its correlation with ventricular function. Using a comprehensive literature search strategy, this review identified all pertinent studies evaluating the role of BNP and NT-proBNP in patients with sepsis. Specifically, adult human studies were reviewed and organized into a narrative style to describe the role of natriuretic peptides in septic cardiomyopathy, fluid resuscitation and outcomes and the prognosis of septic patients.

## BIOCHEMISTRY AND PATHOPHYSIOLOGY

**Figure 1:**
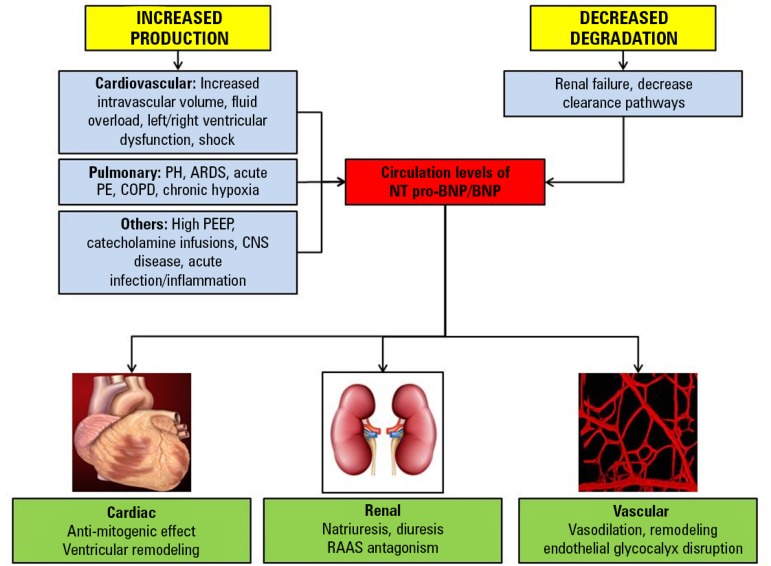
Mechanisms of release and action of natriuretic peptides. PH - pulmonary hypertension; ARDS - acute respiratory distress syndrome; PE - pulmonary embolism; COPD - chronic obstructive pulmonary disease; BNP - B-type natriuretic peptide; NT pro-BNP - N-terminal pro-B-type natriuretic peptide; PEEP - positive end-expiratory pressure; CNS - central nervous system; RAAS - renin-angiotensin-aldosterone system.

B-type natriuretic peptide was first isolated from the porcine brain but was subsequently noted to be secreted by human ventricular myocardial cells.^(23)^ The half-lives of BNP and NT-proBNP in plasma are 22 and 120 minutes, respectively.^(24)^ These natriuretic peptides are predominantly released in response to volume overload and myocyte stretching.^(25)^ B-type natriuretic peptide elimination occurs through several pathways, including natriuretic peptide receptor-C that clears natriuretic peptides from the circulation through receptor-mediated internalization and degradation and less so by neutral endopeptidases through the liver, lung, and kidney in its active form, whereas NT-proBNP is exclusively excreted by the kidneys.^(26-29)^ The release of BNP and NT-pro BNP in sepsis is stimulated by multiple factors (Figure 1). Myocytic stretch with ventricular dysfunction and proinflammatory molecules, such as lipopolysaccharides, interleukin-1, C-reactive protein, and cardiotrophin-1, promote BNP gene expression and release in patients with sepsis.^(30-32)^ Additionally, concomitant renal failure and processes of care such as catecholamine infusions and volume resuscitation lead to an elevation of BNP/NT-proBNP levels.^(33,34)^ In addition to the primary septic process, often pulmonary pathology and interventions such as acute respiratory distress syndrome, chronic obstructive pulmonary disease and mechanical ventilation influence the BNP levels in this population.^(33,35)^ Along with increased production/secretion of BNP, sepsis alters the clearance of BNP due to renal failure and other mechanisms.^(34,36)^

## NATRIURETIC PEPTIDES AND SEPTIC CARDIOMYOPATHY

### Left ventricular systolic dysfunction

Left ventricular (LV) dysfunction is noted variably in 20 - 50% of septic patients, which is likely due to differences in the timing of performing echocardiography and the severity of sepsis among these patients.^(37-39)^ Multiple studies have shown a reduced left ventricular ejection fraction (LVEF) in sepsis, although a recent meta-analysis did not demonstrate any correlation between LV systolic function and LV dimensions with mortality in this population.^(40)^ Formal echocardiography requires expert acquisition and interpretation that limits its generalizability and so a biomarker to detect early LV dysfunction could, therefore, be valuable for evaluating cardiac dysfunction in sepsis.

Charpentier et al. first correlated BNP and LV dysfunction in septic patients, showing that patients with LV fractional area change < 50% and a higher end systolic LV diameter on day two had higher BNP levels than patients with a normal LV fractional area change.^(30)^ Klouche et al. corroborated this finding at days three and four after admission, and patients with LV systolic dysfunction had significantly higher BNP values.^(41)^ In a cohort of cancer patients with sepsis, BNP on day two correlated with patients developing echocardiography-detected LV systolic dysfunction.^(42)^ In contrast to these studies that evaluated single measurements, Post et al. assessed BNP trends, demonstrating an inverse correlation between BNP and LV systolic dysfunction in patients with septic shock.^(31)^ Despite these correlations, it is important to note that echocardiography derived LVEF in sepsis should be interpreted with caution since it is a function of preload, afterload, and myocardial contractility, all of which vary in septic patients.^(43)^ Left ventricular stroke work index, a pulmonary artery catheter-derived parameter, may be a less load-dependent parameter of LV contractility and has been shown to be indicative of myocardial dysfunction in septic shock.^(44,45)^ There are limited data in the current era due to decreased use of pulmonary artery catheters.^(46,47)^

Other authors have shown that BNP levels are elevated in severe sepsis and septic shock regardless of the presence or absence of LV dysfunction.^(48)^ Papanakolaou et al. demonstrated that LVEF was not an independent predictor of BNP elevation in sepsis patients; rather, the severity of the illness seemed to be the primary determinant of BNP elevation.^(49)^ Several other authors also demonstrated elevated BNP levels in septic patients despite normal LV function.^(32,50)^

In summary, BNP/NT-proBNP exhibits a strong correlation with LV function. However, the measurement of LV function in sepsis remains an ongoing debate,^(51,52)^ and thus the correlation ability of BNP is subject to variability. Use of advanced imaging modalities such as tissue Doppler imaging, global longitudinal strain, and speckle tracking echocardiography may be more useful to evaluate the role of myocardial stretching in these patients that results in BNP release.^(34)^

### Left ventricular diastolic dysfunction

Left ventricular diastolic dysfunction is increasingly recognized in modern practice and is noted to have a 20 - 57% prevalence in septic patients.^(53)^ Diastolic dysfunction can potentially explain the etiology of troponin release in sepsis;^(17)^ however, similar mechanistic studies about the role of BNP in sepsis are not readily available. Elevated filling pressures in LV diastolic dysfunction may induce BNP release, and the prior literature has demonstrated that natriuretic peptides are typically lower in patients with preserved LVEF.^(54)^ Patients with LV diastolic dysfunction often have concomitant respiratory failure, necessitating the use of mechanical ventilation, which results in higher levels of BNP release.^(17,35,55,56)^ Several studies have evaluated the correlation of BNP with diastolic dysfunction in septic patients, although they included small numbers of patients, and diastolic function was mainly reported as an ancillary outcome.^(57,58)^ Sturgess et al. demonstrated diastolic dysfunction to be an independent predictor of BNP concentration in septic shock patients, and Lin et al. showed that BNP correlated with the severity of LV diastolic dysfunction in septic patients.^(57,58)^ In contrast, in a study evaluating BNP trends in severe sepsis and septic shock patients, Mclean et al. revealed no apparent correlation between BNP and tissue Doppler velocities of diastolic function (p = 0.15).^(48)^ Based on the currently available data, it appears that BNP/NT-proBNP levels correlate with diastolic dysfunction. Further study of the BNP trends in diastolic dysfunction during sepsis is needed to evaluate its potential role in this increasingly recognized patient population since diastolic dysfunction has demonstrated strong correlations with clinical outcomes.^(17,53)^

### Right ventricular dysfunction

Right ventricular (RV) dysfunction in sepsis and septic shock occurs in 30 - 60% of the population and is associated with worse short and long-term outcomes including mortality.^(17,55)^ In sepsis, in addition to direct myocardial toxicity, increased nitric oxide, acute respiratory distress syndrome, hypoxemic pulmonary vasoconstriction and pulmonary hypertension contribute to worsening RV function.^(55,59-61)^ BNP and NT-proBNP have been described in other disease states that can lead to acute RV failure, including acute respiratory distress syndrome, pulmonary embolism, and chronic obstructive pulmonary disease.^(35,62,63)^ However, the specific role of BNP and NT-proBNP in sepsis-related RV dysfunction remains to be clarified.^(42,48,49)^ McLean et al. demonstrated that BNP elevations in patients with severe sepsis and septic shock correlated with RV systolic performance more closely than with LVEF or LV end-diastolic diameter (r = 0.24; p *=* 0.02).^(48)^ Papanikolaou et al. demonstrated that both RVEF and LVEF were significantly correlated with serial BNP values, showing the likely interdependent nature of both chambers in this population.^(49,55)^ B-type natriuretic peptide may play a role in the diagnosis and management of RV dysfunction in sepsis during the acute phase, analogous to high-sensitivity troponin.^(17)^ Importantly, RV dysfunction has shown stronger correlations with clinical outcomes at 6 months and one year, leading us to speculate on the role of long-term cardiopulmonary interactions.^(55,64)^ There might be a role for serial BNP trending analogous to chronic heart failure, which remains an exciting new avenue for clinical and translational research.^(65)^ However, the contemporary data are limited and inadequate to evaluate the correlation between RV dysfunction and natriuretic peptides.

## FLUID THERAPY AND NATRIURETIC PEPTIDES

Early appropriate fluid resuscitation leads to better outcomes in sepsis, and this often requires a delicate balance between under-resuscitation and volume overload.^(66,67)^ Only approximately 50% of unstable septic patients respond to a fluid challenge and identifying fluid responsive patients remains a significant challenge for clinicians.^(68)^ Dynamic measures such as respiratory variation of the inferior vena cava and changes in stroke volume after passive leg raising are more accurate in identifying patients who will increase their cardiac output in response to a fluid bolus.^(69)^

B-type natriuretic peptide and NT-proBNP have been investigated as markers of volume status and fluid responsiveness. In patients with heart failure, BNP has been shown to be a useful marker of volume status, preload, estimated LV filling pressure, pulmonary artery occlusion pressure, and end-diastolic volume.^(70-72)^ However, these data have not been reliably replicated in patients with undifferentiated shock or sepsis (Table 1).^(41,46,47,49,73-78)^ Hartemink et al. showed that a NT-proBNP cutoff of 3467pg/mL in septic patients predicted fluid responsiveness with a sensitivity of 90% and a specificity of 71%.^(79)^ Similarly, Zhang et al. demonstrated a correlation between delta BNP over three days and fluid balance in septic patients (r = 0.63, p < 0.01).^(80)^ In contrast, several authors showed no correlation between BNP and various parameters of volume status. Among surgical ICU patients, in which 42% had severe sepsis and septic shock, BNP did not correlate with circulating blood volume as measured by radioisotope dilution (coefficient of determination of 0.09; p = 0.45).^(81)^ Pirracchio et al. demonstrated that despite BNP > 1000pg/mL, 9 out of 11 patients with septic shock were still fluid responsive.^(36)^ Sturgess et al. showed a weak correlation between BNP and changes in stroke volume in septic shock patients (r = −0.3, p = 0.40).^(82)^ From the existing limited data, BNP does not appear to be a reliable marker of fluid status or responsiveness in septic patients. B-type natriuretic peptide represents a static variable and is unlikely to correctly estimate RV or LV preload conditions that undergo dynamic adaptation in sepsis. Studies incorporating a BNP-directed fluid management protocol may help elaborate on the role of BNP in fluid status determination in sepsis patients.^(80)^

**Table 1:** Studies demonstrating correlations between natriuretic peptides and cardiac filling pressures in critically ill patients

Study	BNP/NT-proBNP	Timing	Central venous pressure	Pulmonary capillary wedge pressure
Klouche et al.^(41)^	BNP	---	r = 0.45, p < 0.0001	-
Witthaut et al.^(46)^	BNP	---	No correlation	-
Ueda et al.^(47)^	BNP	Day 2	r = 0.744, p < 0.01	r = 0.709, p < 0.01
Papanikolaou et al.^(49)^	BNP	Day 1	Correlates with in septic shock	No correlation
Turner et al.^(76)^	BNP	---	r = 0.12; p = 0.12	-
Rivers et al.^(77)^	BNP	12 hours	r = 0.36, p < 0.001	-
Varpula et al.^(78)^	NT-proBNP	Admission	Correlations	Correlates

BNP - B-type natriuretic peptide; NT-proBNP - N-terminal pro-B-type natriuretic peptide.

## NATRIURETIC PEPTIDES AND MORTALITY

B-type natriuretic peptide and NT-proBNP have shown a varied correlation with short-term mortality (Tables 2 and 3).^(30,31,41,42,47,49,57,78,82-90)^ In patients with sepsis and septic shock, Charpentier et al. first showed that a BNP of > 190pg/mL on day two of admission was associated with a 5.7-fold increased risk of death (95% confidence interval [95%CI] 1.8 - 27.5; p = 0.14).^(30)^ Brueckmann et al. evaluated NT-proBNP, also on day two of ICU admission, and noted a NT-proBNP of > 1400pg/mL was associated with a 3.9 times higher risk of mortality (95%CI, 1.6 - 9.7).^(83)^ Since these initial studies, many other investigators have evaluated the correlation of BNP and NT-proBNP with mortality in sepsis in various clinical settings (Tables 2 and 3). More recently, Khoury et al. demonstrated BNP at admission was strongly correlated with in-hospital, 90-day and 60-month mortality.^(87)^ Using predetermined cut-offs, they demonstrated serial increases in BNP to be associated with worse short-term mortality.^(87)^ Similar data have been noted in patients with acute exacerbations of chronic obstructive pulmonary disease, leading to the hypothesis that BNP should be used as a continuous outcome predictor in these patients.^(35)^ Studies in the emergency room have shown higher natriuretic peptide levels to be associated with higher mortality.^(88,89)^ There have been variable cut-offs stated in the literature-Perman et al. demonstrated a BNP > 49pg/mL to be associated with increased mortality while Chen et al. used a cutoff of > 113pg/mL.^(88,89)^ The majority of studies focused on ICU patients, several of which evaluated mechanically ventilated patients. In mechanically ventilated septic patients, primary cardiac dysfunction often complicates the pulmonary process, leading to elevation of BNP via multiple mechanisms as highlighted in figure 1.^(34,35,55)^ In mechanically ventilated patients, both BNP and NT-proBNP have been demonstrated to have a good correlation with mortality with areas under the receiver operating characteristic curve (AUROC) of 0.99 and 0.8, respectively.^(84,90)^ In cancer patients who develop sepsis, NT-proBNP levels of > 6,624pg/mL predicted ICU mortality (AUROC 0.87; 95%CI 0.77 - 0.97; p < 0.001) on day two of ICU admission.^(42)^ Several other studies also reported BNP correlations with mortality at various time points during hospital and ICU stays.^(41,47,77,78,85,89-91)^ Most studies included patients already diagnosed with a sepsis syndrome before inclusion, and therefore limited data are currently available for the association of BNP and outcomes prior to the onset of sepsis. One study evaluated patients who developed sepsis during their ICU stay (evolving sepsis) and demonstrated that BNP measured on day five was correlated with 30-day mortality in these patients.^(31)^

**Table 2:** Studies evaluating N-terminal pro-B-type natriuretic peptide and mortality

Study	N	Septic shock %	Timing of NT-proBNP	Definition of LV dysfunction	Mortality (%)		Optimal cut-off (pg/mL)	AUC
					ICU/hospital	30-day		
Mokart et al.^(42)^	51	100	Day 2	LVEF < 55%	51	---	6,624	0.87
Varpula et al.^(78)^	254	---	Hospital admission	High PCWP quartiles	13/26	---	7,090	0.631
Sturgess et al.^(82)^	21	100		LV stroke work index	2	---	400	0.67
Brueckmann et al.^(83)^	57	0	Day 2	LVEF 35 - 50% - Moderate	---	28	1400	0.68
Roch et al.^(84)^	39	100	Day 2	LVEF < 35% - Severe	56	---	13,600	0.8
Guaricci et al.^(85)^	40	0	Day 3	LVEF < 45%	---	55	1,000	0.99

NT-proBNP - N-terminal pro-B-type natriuretic peptide; LV - left ventricle; ICU - intensive care unit; AUC - area under the curve; LVEF - left ventricular ejection fraction; PCWP - Pulmonary capillary wedge pressure.

**Table 3:** Studies evaluating B-type natriuretic peptide and mortality

Study	N	Septic shock %	Timing of BNP	Definition of LV dysfunction	Mortality (%)			Optimal cut-off (pg/mL)	AUC
					ICU/hospital	30-days	1-year		
Charpentier et al.^(30)^	34	74	Day 2	LVFAC < 50%	-	29	---	190	0.66
Post et al.^(31)^	93	100	Day 5	LVEF < 50%	---	41	---	121	0.65
Klouche et al.^(41)^	47	---	---	LVEF < 45%	28	---	---	---	---
Ueda et al.^(47)^	33	67	Day 2	LV stroke work index	-	39	---	650	0.85
Papanikolaou et al.^(49)^	42	71%	Day 1	LVEF 35 - 50% (moderate) LVEF < 35% (severe)	-	48	---	800	0.70
Sturgess et al.^(82)^	21	100	---	LVEF < 55%	29	---	---	254	0.78
Zhao et al.^(86)^	102	---	< 24 hours	---	---	38	---	681	0.92
Khoury et al.^(87)^	259	---	Admission	---	21	---	---	1,000	0.68
Perman et al.^(88)^	825	---	ED arrival	---	3	---	---	49	0.69
Chen et al.^(89)^	327	0	< 24 hours	---	---	37	---	113	0.74
Yucel et al.^(90)^	40	---	Day 1,2	---	---	50	---	32.1	0.99

AUC - area under the curve; BNP - B-type natriuretic peptide; ED - Emergency Department; ICU - intensive care unit; LV - left ventricle; LVEF - left ventricular ejection fraction; LVFAC - left ventricular fractional area of change; PAWP - pulmonary capillary wedge pressure.

Some studies have shown no correlation between BNP and mortality in sepsis patients. Among forty patients with sepsis and septic shock, Mclean et al. reported that BNP did not predict in-hospital mortality (odds ratio 1.0, 95%CI 0.99 - 1.0).^(48)^ Other authors have also corroborated these findings, including in subsets of severe sepsis and septic shock.^(74,82,92,93)^ These discrepant results may be partly due to the heterogeneity of sepsis, differences in timing of BNP measurement and types of assays used, small sample sizes and lack of controls for septic cardiomyopathy.^(33,51,55)^ The optimal timing for measurement of BNP still remains to be ascertained. In the reported literature, BNP and NT-proBNP were measured anytime between admission and day five.^(22)^ Although a recent meta-analysis demonstrated BNP to be a predictor of mortality in septic patients with pooled sensitivity and specificity of 79% and 60%, there was significant heterogeneity (I^2^ = 64%) among the evaluated studies.^(22)^ In this systematic analysis, BNP assays, clinical endpoints, and vasopressor use varied markedly among the enrolled studies.^(22)^ Additionally, exclusion of pre-existing conditions known to elevate BNP and NT-proBNP differed among studies, with five of the 12 studies including cases with a combination of either pre-existing kidney and/or cardiac disease. Furthermore, two of the largest included studies were performed in the emergency room, representing sepsis patients at a markedly different time of resuscitation than later in their hospital course.^(88,89)^

Despite suggestions of a correlation with mortality, the role of BNP in mortality outcomes and prognosis during sepsis needs further evaluation in larger prospective studies. A single marker known to be elevated in a wide range of pathophysiologic states is unlikely to be a perfect fit as a prognostic marker in sepsis.^(33)^

### Scoring systems, optimal cut-offs and serial testing

In recent years, some authors have used natriuretic peptides to develop novel scoring systems or demonstrate more accurate prognostic capacities than pre-existing scoring systems.^(87,94)^ Khoury et al. demonstrated that BNP at admission was more predictive of short-term mortality than the Sequential Organ Failure Assessment (SOFA) score.^(87)^ In contrast, Ryoo et al. demonstrated a combination of BNP with the SOFA score resulted in better outcomes and prognosis in septic patients who either method independently.^(95)^ In surgically critically ill patients, the use of a bioscore combining BNP with lymphocyte percentage and procalcitonin showed a strong prediction for sepsis onset in this population.^(94)^

The optimal cutoff of BNP and NT-proBNP to predict mortality in sepsis remains uncertain and varies between 32 to 681pg/mL for BNP and 400 to 13,600pg/mL for NT-proBNP. A recent meta-analysis was unable to determine optimal cutoffs for mortality outcomes and prognosis in patients with sepsis.^(22)^ In the evaluation of patients with dyspnea, a BNP level of < 100pg/mL has been used as a sensitive and specific value for ruling out heart failure. A level of > 400pg/mL suggests that heart failure is potentially a contributor to the patient's symptoms with 90% specificity.^(96)^ For NT-proBNP, a cutoff of 300pg/mL is used to rule out heart failure, whereas optimal 'rule-in' cutoffs vary, depending on age (450pg/mL for < 50 years, 900pg/mL for 50 - 75 years, and 1800pg/mL for > 75 years).^(96-98)^

Akin to acute heart failure, there may be utility in trending natriuretic peptides in patients with sepsis.^(65)^ Serial BNP testing may have greater clinical utility in outcomes and prognosis for patients with sepsis rather than as a one-time measure. Papanikolaou et al. recently demonstrated that persistently elevated BNP > 500pg/mL was a better predictor of 28-day mortality than isolated BNP values.^(49)^ Inability to reduce BNP to < 500pg/mL predicted 28-day mortality with AUROC 0.74 (95%CI 0.55 - 0.93; p = 0.03).^(49)^ Similarly, several other authors showed that improvements in BNP in serial measurements conducted over their ICU stay were associated with better survival, and variation between baseline BNP compared to 72 hours was significantly associated with 28-day mortality.^(41,85)^ 'Delta BNP' is a promising dynamic marker used to assess cardiovascular function and the outcomes of sepsis.

## SPECIAL CONSIDERATIONS

### Impact of renal failure, age, and sex

Kidney dysfunction is a well-known cause of elevated natriuretic peptide levels and often confounds assessment in sepsis where AKI is seen in nearly 50 - 65% of patients.^(34,99)^ An inverse correlation has been shown between BNP and kidney dysfunction in critically ill patients. However, optimal cutoffs are yet to be defined in the septic population.^(22,74,75)^ Several studies have shown elevated BNP levels in kidney dysfunction compared to patients with normal renal function despite similar cardiac function and hemodynamics.^(36,100)^ Abnormalities in kidney function in sepsis remain a major confounder in studies evaluating the prognostic value of BNP in sepsis, as current studies variably exclude pre-existing chronic kidney disease and inconsistently adjust for acute kidney injury in the analysis.^(22)^ In septic patients, studies have shown conflicting results regarding correlations between BNP and serum creatinine; Roch et al.^(84)^ showed a weak correlation (r = 0.2, p = 0.03), whereas Ueda et al.^(47)^ showed no correlation. In addition to renal function, age and sex influence natriuretic peptide levels. Increasing age is known to cause elevated BNP and NT pro-BNP levels, although the mechanisms remain under investigation.^(100,101)^ Women generally have higher levels of BNP and NT pro-BNP due to higher estrogen levels.^(101)^ Cutoffs based on age and sex have been suggested in primary care patients and heart failure populations but have not been validated in sepsis populations at the current time.^(102)^

## CONCLUSIONS

Natriuretic peptides are commonly elevated in patients with sepsis. Despite suggestions that their elevation can predict mortality and other clinically meaningful outcomes, studies are conflicting, and the role of these biomarkers remains unclear. The role of BNP and NT-proBNP in evaluating acute and chronic cardiovascular morbidity in sepsis and septic shock remains an exciting new avenue, and further research is warranted. Trends rather than isolated values may be more useful, and their use in conjunction with other clinical data may define a clearer role for BNP and NT-proBNP in sepsis.
